# The ethnoprimatology of the Maijuna of the Peruvian Amazon and implications for primate conservation

**DOI:** 10.1186/s13002-018-0207-x

**Published:** 2018-03-07

**Authors:** Carla Mere Roncal, Mark Bowler, Michael P Gilmore

**Affiliations:** 1 Environmental Science and Policy, George Mason University, 4400 University Drive, Fairfax, VA 22030 USA; 2 Institute for Conservation Research, San Diego Zoo Global, 15600 San Pasqual Valley Road, Escondido, CA 92027 USA; 3 School of Integrative Studies, George Mason University, 4400 University Drive, Fairfax, VA 22030 USA

**Keywords:** Ethnoprimatology, Maijuna, Indigenous people, Primates, Peruvian Amazon

## Abstract

**Background:**

In Amazonia, primates are not only an important food source but they also hold significant cultural and symbolic value for many indigenous groups. We document the relationship between primates and community members of the Maijuna indigenous community of Sucusari in the Peruvian Amazon and describe how ethnoprimatological studies provide a better understanding of the significance of primates in people’s lives. Additionally, we explore how ethnoprimatological studies can help inform and enhance primate conservation initiatives.

**Methods:**

Semi-structured interviews were conducted with 50 residents of the community of Sucusari to assess the classification, cultural significance and traditional uses, beliefs, ceremonies and stories of primates within the Sucusari River basin.

**Results:**

Primates play an important role in the lives of individuals in the Sucusari community. They are distinguished by their arboreal lifestyle, and among the 11 species reported in the area, seven (*Lagothrix lagotricha*, *Alouatta seniculus*, *Pithecia monachus*, *Callicebus* spp., *Saimiri sciureus*, *Leontocebus nigricollis*) are highly recognized and culturally salient. Primates are used as food, medicine, pets, domestic tools and in the production of handicrafts. They are primarily hunted for local consumption, with larger primates such as *L*. *lagotricha* being preferred. *Lagothrix* *lagotricha* was also the most commonly reported pet species and the only observed pet primate in the community during surveys. Maijuna traditional beliefs include ancestral dietary taboos for *A*. *seniculus*, which are referred to as sorcerer monkeys, but this taboo is no longer fully adhered to. Maijuna traditional stories associated with primates describe the origin of primates found in Sucusari.

**Conclusion:**

Primates are embedded in the intricate sociocultural system of the community of Sucusari. Better understanding the relationship between primates and people can help to focus conservation efforts on primate species of particularly high sociocultural importance as well as ecological value, such as *L. lagotricha*. We highly recommend the inclusion of ethnoprimatological studies into primate conservation initiatives to accomplish more effective conservation planning, ultimately integrating the goals of biodiversity conservation with the cultural and economic needs of indigenous and local communities.

## Background

Neotropical forests contain the highest diversity of primate fauna in the world with 171 out of 504 primate species listed in the most recent taxonomic compilation, followed by Asia (119 species), Africa (111 species), and Madagascar (103 species) [1]. Forest sites in Amazonia typically contain ten or more sympatric monkey species, comprising a large part of the local arboreal vertebrate biomass [2, 3]. Moreover, they are important seed dispersers and predators [4], playing a key role in forest dynamics [5–8]. Amazonian primate populations are threatened by deforestation and forest degradation [9–12] and by widespread hunting [13, 14].

Primate populations and their fates are inevitably linked to the activities of resident human populations. While a number of studies examine the effects of human activities on primate populations [15–17], few studies in Amazonia have recognized the importance of studying the interconnection between humans and other primates, from the perspective of local human residents [18–21]. Ethnozoology has become an important field that is focused on the interactions between humans and animals encompassing a wide array of studies. Ethnozoological research has demonstrated that human populations possess extensive ecological knowledge about animals that can complement western scientific knowledge as well as provide valuable data for conservation [22]. Ethnoprimatology, an important subdiscipline of ethnozoology, is focused on the interactions between humans and non-human primates where humans are considered an integral part of primate ecosystems [23]. Some ethnoprimatological studies in the Amazon have demonstrated the significant role of primates in the lives of indigenous people [18–21, 24, 25], encompassing subsistence hunting, food preference and avoidance, beliefs, ceremonies, and stories that define the symbolism and identity of indigenous groups. Primates are therefore embedded within complex sociocultural relationships, where human hunting, traditional uses, beliefs, and myths affect primate ecology. Hence, ethnoprimatological studies provide valuable insights and information regarding the cultural significance of primates to indigenous peoples that directly effects the development and implementation of sustainable management practices and conservation strategies.

**Fig. 1:**
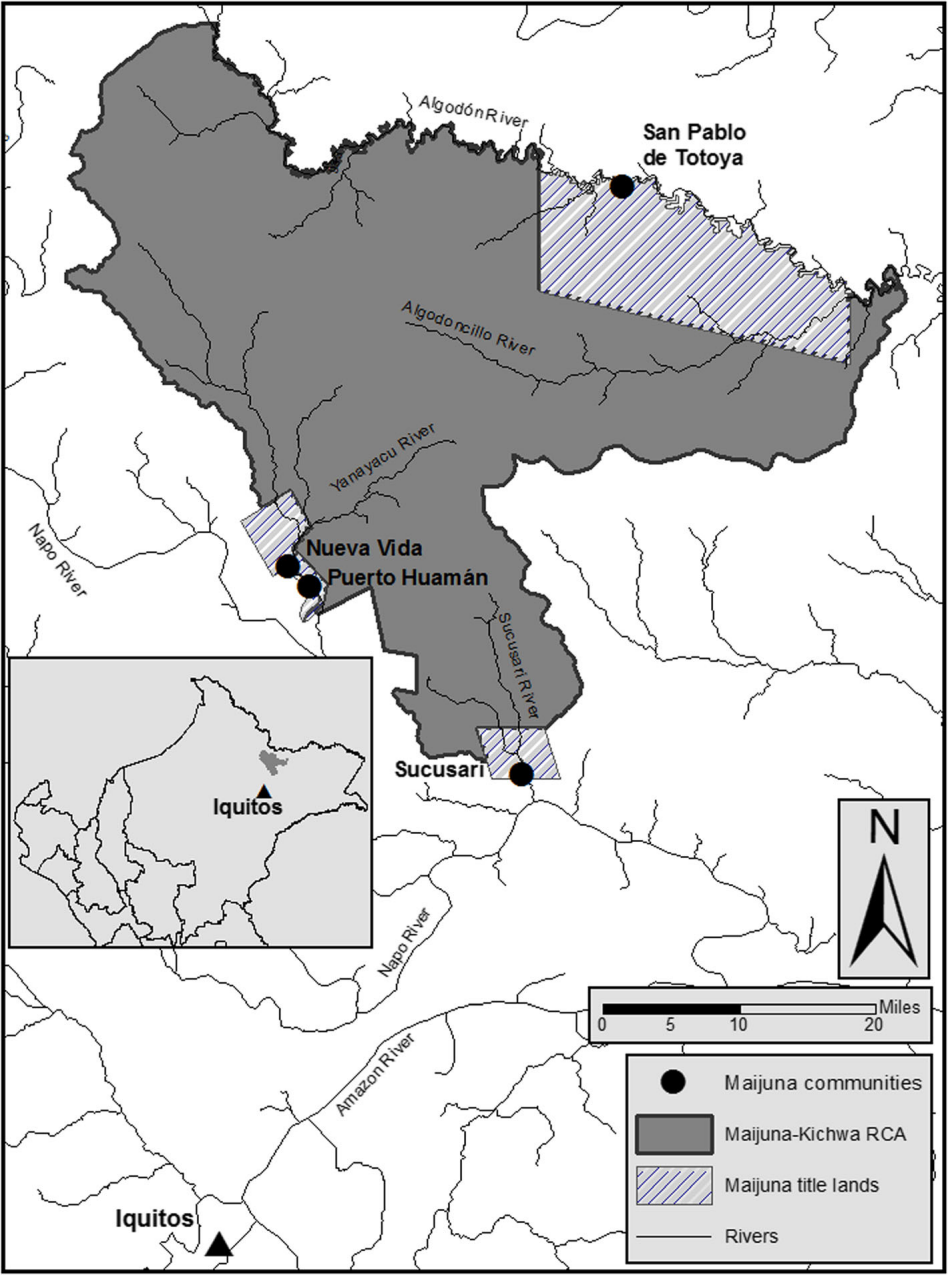
Study area—the Maijuna community of Sucusari, Loreto, Peru

The Maijuna (also known as the Orejón or Coto) are a Western Tucanoan people who live in the northeastern Peruvian Amazon [26]. Studies on the traditional knowledge of the Maijuna have focused on ethnobotanical information [26–28], yet no research has been conducted in regards to the sociocultural importance of primate populations to the Maijuna indigenous group. Currently, less than 500 Maijuna individuals live within Maijuna ancestral territory, distributed in four communities: Puerto Huamán and Nueva Vida along the Yanayacu River, San Pablo de Totolla (Totoya) along the Algodón River, and Sucusari along the Sucusari River (Fig. 1). Each community is recognized as a native community (*Comunidad Nativa*) by the Peruvian government and has been granted title to the land surrounding their community [29].

Over the years, Maijuna ancestral lands have been subjected to many illegal and unsustainable activities by outsiders causing an overexploitation of natural resources and negatively impacting local livelihood strategies [30]. In 2006, the Maijuna decided to push for the formal protection of their lands in the form of an official protected area to prevent further degradation of their territory and biocultural resources. Since then, the Maijuna have increased their interest in developing sustainable activities to protect their natural resources [31]. In June of 2015, after almost 10 years of struggle by the Maijuna and their allies, the National Government of Peru formally created the Maijuna-Kichwa Regional Conservation Area (MKRCA). The Decree No. 008-2015 of the Ministry of Environment officially protects 391,039 ha of Maijuna ancestral territory to conserve its vast biodiversity and support local livelihoods [32]. Thus, identifying and understanding the main drivers of anthropogenic pressures on wildlife populations is critically important to develop effective management plans in the new MKRCA. Within this framework, the present study aims to document and understand the significance and relationship between primates and community members of the Maijuna indigenous community of Sucusari. Specifically, it described and assessed the cultural significance and traditional uses, beliefs, ceremonies and stories of primates in the Sucusari River basin, with the ultimate aim to better inform primate conservation.

## Methods

### Study area

This study was conducted in the Maijuna indigenous community of Sucusari, situated along the Sucusari River, a tributary of the Napo River, in northeastern Peru (Fig. 1). The community is located approximately 126 km by river from Iquitos, the capital of the Department of Loreto [27]. The Sucusari community has legal title to 4771 ha, which adjoins the recently established MKRCA, and is the only village located within the Sucusari River basin. An ecotourism lodge, established in 1983, is located approximately 4.5 km downriver from the main community [33].

The community has 166 residents divided into 32 monofamilial or plurifamilial houses. Within the total number of inhabitants of the Sucusari community, 59% are Maijuna, 35% are mestizos,^1^ and the remaining 6% are Kichwa. The main subsistence activities of community members include hunting, fishing, swidden-fallow agriculture, and the gathering of various forest products [30]. To generate income, residents sell game meat, domesticated animals, agricultural products, and a variety of non-timber forest products [30].

The MKRCA harbors a vast area of intact rain forest, encompassing the headwaters of seven rivers (one of which is the Sucusari River) that supply two large Amazonian watersheds (i.e., Napo and Putumayo). The area also contains a complex of high terraces—a previously unknown habitat—that shelters a flora and fauna with a number of new, rare, and specialized species [32]. The MKRCA harbors biological resources that are vital for the survival of local people, including non-timber forest products (e.g., *Mauritia flexuosa* palm fruits), birds, tortoises, fish (e.g., *Arapaima gigas*, *Osteoglossum bicirrhosum*), and large mammals such as primates [32]. Within the study area, 11 primate species have been previously reported in the literature [30, 34–36] (Table 1).

**Table 1:** Primate species reported in the Sucusari River basin or surrounding areas, Napo River, Peru

Species^*^	Mass (kg)	English common name	Local name	Citations
*Cebuella pygmaea*	0.1–0.14	Pygmy marmoset	Leoncito	[32, 38]
*Leontocebus nigricollis*	0.4–0.5	Black mantle tamarin	Pichico	[32, 38–40]
*Aotus vociferans*	0.7–1.2	Spix’s night monkey	Musmuqui	[32, 38, 40]
*Saimiri sciureus*	0.6–1.4	Common squirrel monkey	Fraile	[32, 38, 40]
*Callicebus discolor*	0.9–1.4	Red titi	Tocón cenizo/colorado	[32, 38, 40]
*Callicebus lucifer*	0.8–1.5	Lucifer titi	Tocón negro	[32, 38, 40]
*Pithecia monachus*	2.2–2.5	Monk saki	Huapo	[32, 38, 40]
*Sapajus macrocephalus*	1.2–3.6	Large-headed capuchin	Mono/machín negro	[32, 38, 40]
*Cebus albifrons*	1.7–4.5	White-fronted capuchin	Mono/machín blanco	[32, 38, 40]
*Lagothrix lagotricha*	3.6–10	Common woolly	Mono choro	[32, 38–40]
*Alouatta seniculus*	3.6–11.1	Red howler	Mono coto/colorado/brujo	[32, 38–40]

^*^Species listed in order of increasing body mass [76]

### Data collection

We interviewed 50 residents of the Sucusari community in August 2014, targeting both male and female adults (> 18 years old) that were available and willing to be interviewed. The sample included 30 (60%) males and 20 (40%) females, with a mean age of 43.58 (SD ± 13.82) years (Table 2). For the purposes of this project, hunters were defined as any individual of the community that has hunted once or more during their lifetime. Individual interviews were conducted in Spanish or in Maijuna, the latter with the help of a community leader that is fluent in both languages. Before beginning the interviews, prior informed consent (PIC) was obtained from the Sucusari community as well as from individual research participants [37].

**Table 2:** Demographics of the interviewees from the Maijuna community of Sucusari, Loreto, Peru

Characteristics	
Sample (*N*)	50
Gender	
Female	20 (40%)
Male	30 (60%)
Ethnicity	
Maijuna	26 (52%)
Mestizo	23 (46%)
Kichwa	1 (2%)
Age (years)	
Mean (SD)	43.58 (13.82)
Min–max	21–72
Years of education	
Mean (SD)	5.36 (3.35)
Min–max	0–13
Years living in Sucusari	
Mean (SD)	25.26 (22.49)
Min–max	0.5–69

Individuals were interviewed using a semi-structured questionnaire [38] to gather sociocultural information (i.e., uses, beliefs, ceremonies, and stories) for all primate species found within the Sucusari River basin. The beginning of the questionnaire contained basic demographic questions and then shifted to questions focused on how individuals define and classify monkeys. This was then followed up by a freelisting exercise to determine the cultural salience of the primate species found in the area. To verify local knowledge, the questionnaire then included photographs of 20 primate species, nine of which do not occur in the area serving as a control. The control species were *Saguinus mystax* [39], *Leontocebus tripartitus* [39], *Aotus nigriceps* [40], *Callicebus cupreus* [41], *Saimiri boliviensis* [42], *Cacajao calvus* [43], *Ateles belzebuth* [44], *Ateles chamek* [44], and *Lagothrix poeppigii* [42]. The remaining 11 photographs were of the species reported to occur in the area [30, 34–36] and were used to validate the supposition that people were aware of the species in their area, rather than guessing. The 11 primate species were: *Cebuella pygmaea* [42], *Leontocebus nigricollis* [39], *Aotus vociferans* [40], *Saimiri sciureus* [42], *Callicebus discolor* [41], *Callicebus lucifer* [41], *Pithecia monachus* [45], *Cebus albifrons* [42], *Sapajus macrocephalus* [46], *Alouatta seniculus* [42], and *Lagothrix lagotricha* [42]*.* Questions regarding the use, beliefs, ceremonies, and stories for the different primate species reported in the area were then only asked when individuals were able to correctly identify the primate as well as correctly indicate its occurrence in the area (hereafter described as the “correct location”).

### Data analysis—freelisting and cultural salience

Freelisting is a method that can be used to determine the cultural salience of named species, representing the cultural importance of a particular animal within the studied community [43]. Freelisting was used at the beginning of interviews to determine the cultural salience of the primate species found in the area. Cultural salience calculations assume that (1) items named by most individuals are more salient and (2) the first items in the list are more salient [47]. Based on these assumptions, Quinlan [48] created the following formula to determine the cultural salience of each animal species listed by an individual:

Salience = (1 + *length*_*i*_ − *position*_*i*_)/*length*_*i*,_ where *length* is the total number of animals listed by individual *i* and *position* is the location of a particular animal in the list of individual *i*. Animals not listed by an individual had a cultural salience of zero.

The cultural salience of each animal was calculated using the following equation:

Cultural salience = Σ *salience*_*i*_/*n*, where: *n* (*n* = 50) is the total number of respondents who participated in the study.

## Results

### Defining and classifying monkeys

In order to elucidate how primates are locally defined and classified within the Sucusari community, interviewees were asked to define the term *mono* in Spanish, which means monkey in English. There was no clear consensus of the definition and classification of primates. Responses given were coded and divided into eight categories: (1) live in trees (68%), (2) jump/climb on branches (52%), (3) eat fruits/food in trees (32%), (4) possess a long tail (32%), (5) possess a prehensile tail (12%), (6) can go down to the ground to eat (10%), (7) distinct fur (4%), and (8) live in various places (2%). Most of these qualities also apply to some of the non-primate species listed by respondents during freelisting, such as Procyonidae *Potos flavus*.

### Freelisting and cultural salience

During the beginning of the interviews, individuals were asked to freelist all of the monkey species that are present in the Sucusari River basin. The interviewees listed 11 primate species in total. Five species were mentioned by more than 80% of respondents: *Pithecia monachus* (96%), *Saimiri sciureus* (94%), *Alouatta seniculus* (92%), *Lagothrix lagotricha* (90%), and *Leontocebus nigricollis* (84%). Additional species listed were *Cebus albifrons* (72%), *Aotus vociferans* (50%), *Cebuella pygmaea* (40%), and *Sapajus macrocephalus* (10%). The titi monkeys (*Callicebus discolor* and *Callicebus lucifer*) represented a unique case. Although 92% of the respondents mentioned titi monkeys during the freelisting, 61% referred to them with a single common name (*tocón*) and, therefore, did not differentiate between the two species. It is also worth noting that during freelisting, 22% of respondents listed only primate species whereas 78% of them also mentioned other non-primate mammal species (Table 3). The kinkajou (*Potos flavus*), a nocturnal mammal species with many features that resemble a primate, was mentioned by 64% of all respondents.

**Table 3:** Non-primate mammal species reported as monkeys during freelisting

Scientific name	English common name	Local name	Number of respondents	Percentage of respondents
*Potos flavus*	Kinkajou	Chosna	32	64
*Sciurus* sp.	Squirrel	Ardilla	15	30
*Coendou* sp.	Porcupine	Cashacushillo	13	26
*Nasua nasua*	Coati	Achuni	12	24
*Choloepus* spp*.*	Sloth	Pelejo	11	22
*Tamandua tetradactyla*	Tamandua	Shiwi	8	16
*Eira barbara*	Tayra	Manco	6	12
*Didelphis marsupialis*	Common opposum	Zorrito	1	2

**Fig. 2:**
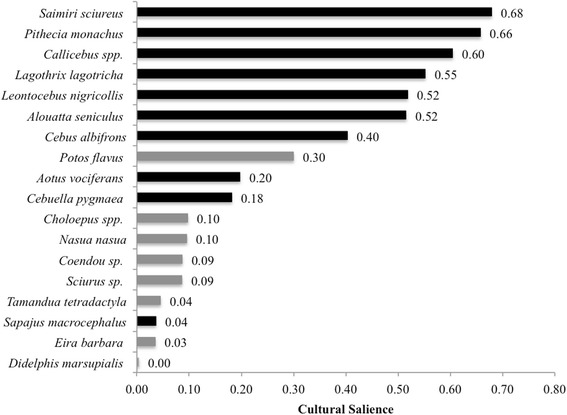
Cultural salience of all primate and non-primate species listed by interviewees during freelisting. Darker and lighter bars are primate and non-primate mammal species, respectively. Cultural salience ranges from 0 to 1, with 1 being the most culturally salient

Both primate and non-primate mammal species mentioned during the freelisting were included to calculate the cultural salience of the animals listed. Analysis showed that the most culturally salient primate species were *S*. *sciureus* (0.68), *P*. *monachus* (0.66), *Callicebus* spp*.* (0.60), *L*. *lagotricha* (0.55), *A*. *seniculus* (0.52), and *L*. *nigricollis* (0.52). The species with lower cultural salience were *C*. *albifrons* (0.40), *A*. *vociferans* (0.20), *C*. *pygmaea* (0.18), and *S*. *macrocephalus* (0.04) (Fig. 2). Given that 61% of interviewees referred to the titi monkeys using a single common name (*tocón*) and did not differentiate between the two species (*C*. *discolor* and *C*. *lucifer*), the single common name was used for the analysis (*Callicebus* spp*.*). *P*. *flavus* had the highest cultural salience (0.30) among non-primate mammal species, even greater than some primate species.

**Fig. 3:**
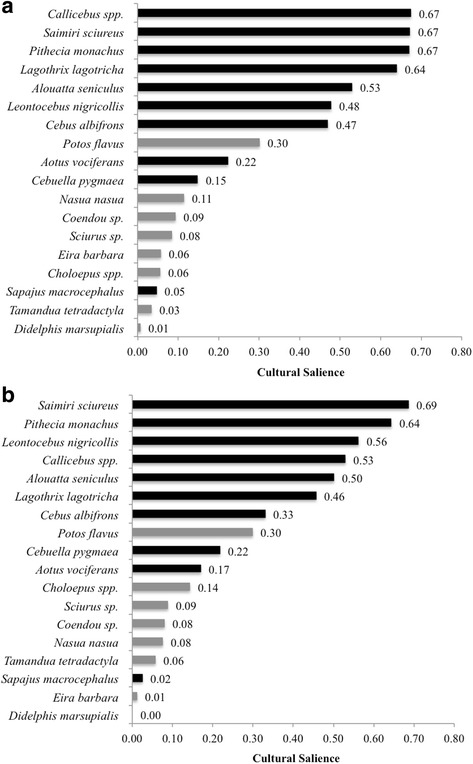
Cultural salience of primate and non-primate species for Maijuna (**a**) and non-Maijuna (**b**) interviewees. Darker and lighter bars are primate and non-primate mammal species, respectively. Cultural salience ranges from 0 to 1, with 1 being the most culturally salient

It is important to note that members of the Sucusari community can be divided into two main groups based on ethnicity: Maijuna and non-Maijuna individuals, the latter including mestizos and Kichwa individuals. Indigenous and non-indigenous people have different cultural perceptions, traditions, and views regarding the environment that surrounds them [49, 50]. For this reason, the cultural salience of freelisted species was calculated for each group separately (Fig. 3a, b). Seven primate species (*S*. *sciureus*, *P*. *monachus*, *Callicebus* spp., *L*. *nigricollis*, *L*. *lagotricha*, and *A*. *seniculus*) had the highest cultural salience for both groups. However, for the Maijuna, *L*. *lagotricha* had a cultural salience of 0.64, almost 20% higher than for non-Maijuna respondents (0.46).

### Photo identification exercise

**Fig. 4:**
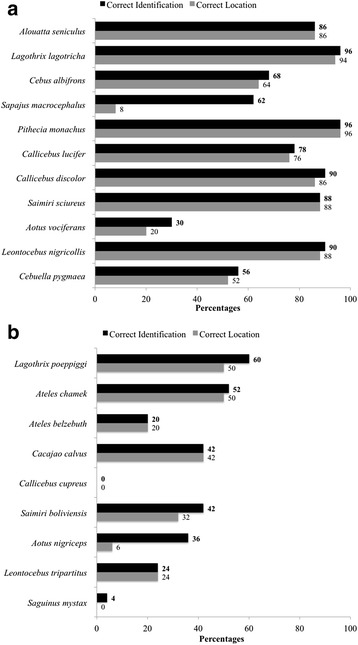
Percentages of correct identification and location of primates reported along the Sucusari River (**a**) and control species (**b**). Correct identification (darker bars) and location (lighter bars) of primates were assessed through the photo identification exercise

Seven out of the 11 primate species found within the Sucusari River basin were correctly identified during the photo exercise by more than 75% of the interviewees (Fig. 4a). These species include *L*. *lagotricha* (96%), *P*. *monachus* (96%), *C*. *discolor* (90%), *L*. *nigricollis* (90%), *S*. *sciureus* (88%), *A*. *seniculus* (86%), and *C*. *lucifer* (78%). These were also the primate species with the highest cultural salience (Figs. 2 and 3)*.* In regards to indicating the correct location of each primate species, respondents who correctly identified the primate species from a photo were more likely to correctly identify its occurrence in the area. The exception to this was *S*. *macrocephalus*, which was considered to be present in the Sucusari River basin by only 8% of the respondents (Fig. 4a). It is worth noting that the species that were most commonly confused by the respondents during the photo exercise were *S*. *macrocephalus* and *C*. *albifrons*, and they had the third (62%) and fourth (68%) lowest rates of identification by interviewees, respectively. The two species with the lowest identification rates were *A*. *vociferans* (30%), which is the only nocturnal primate in the area, and *C*. *pygmaea* (56%), which is the smallest primate of the group and cryptically colored. As expected, the control species were not recognized by the majority of interviewees, except for *L*. *poepiggi*, identified as *choro colorado* in Spanish or red woolly monkey in English, and *A*. *chamek*, identified as *mono araña* in Spanish or spider monkey in English, by 60 and 52% of the interviewees respectively (Fig. 4b). These two species are some of the largest primates and highly consumed by Amazonian indigenous peoples.

### Traditional uses of primates

Interviewees that correctly identified a primate in the photo exercise as well as correctly indicated its occurrence in the Sucusari River basin were asked to list all the known uses for that primate species. Among all responses given, primates are used as food, medicine, pets, and domestic tools (e.g., tails used as dusters to clean houses) and in the production of handicrafts (Table 4). Although it was not part of the questionnaire, some respondents provided further information regarding primate food preparation, and this information was augmented through direct observation. After primates are killed by hunters using shotguns, their fur is burned off over an open fire, constantly being flipped to prevent burning. The entrails of the monkey are then removed and it is butchered. The meat is then cooked by grilling, boiling, or smoking, and all edible parts are then consumed. No information on specific primate species or body parts consumed by specific members of the populace was indicated nor observed, and detailed recipes were not obtained through the questionnaire. However, three respondents reported that *L*. *lagotricha* is considered the preferred primate species to hunt, not only for its larger size but also for its flavor. Hunting and eating woolly monkeys (*L*. *lagotricha*) is especially preferred during the fruiting season of *leche huayo* (*Couma macrocarpa*), also called *leche caspi*, due to their increased body fat content during that period.

**Table 4:** Traditional Maijuna and Kichwa uses, beliefs, ceremonies, and stories for the primates of Sucusari, Loreto, Peru

Species	Maijuna name^a^	Kichwa name^b^	Use	Beliefs/ceremonies	Story
*Cebuella pygmaea*	Camishishi	Chambirisho	Pet	–	No
*Leontocebus nigricollis*	Chichi	*–*	Edible^c^; pet	–	Yes^g^
*Aotus vociferans*	ɨtɨ	Makuru	Edible^c^; pet	Devil monkey	Yes^g^
*Saimiri sciureus*	Bo chichi	Barisa	Edible; pet; bones used as needles to sew handicrafts; teeth used in handicrafts	They are considered the father-in law of the white-fronted capuchin, which is the reason why they travel together in the same troop^g^	Yes^g^
*Callicebus discolor*	Ñame bao	Sukali	Edible; pet; tail used as a duster; bones used as needles to sew handicrafts	–	Yes^g, e^
*Callicebus lucifer*	Bao	Yana-sukali	Edible; pet; tail used as a duster	–	Yes^g^
*Pithecia monachus*	Baotutu	Parahuaco	Edible^d^; pet; tail used as a duster, decoration, and to treat digestive disorders^e^; bones used as needles to sew handicrafts	Poisonous for dogs^g^	Yes^g^
*Sapajus macrocephalus*	Nea taque	*–*	Edible; pet	–	No
*Cebus albifrons*	Bo taque	*–*	Edible; pet; bones and fur used for handicrafts	–	Yes^g^
*Lagothrix lagotricha*	Naso	Arawata	Edible^d^; pet; commercialization of bushmeat^f^	Used in the ceremony of the first yearly harvest of *Bactris gasipae* (pijuayo) palm fruits (last ceremony was performed in 1974)^g^	Yes^g^
*Alouatta seniculus*	Jaiquɨ	Imú	Edible; pet; bones used as needles to sew handicfrafts; hyoid bone used to drink water^g^, in handicrafts, or for soar throats^g^; fur is used to cover drums^e^; commercialization of bushmeat^g^	Sorcerer monkey^g^	Yes^g, e^

^a^Transcription of Maijuna words was accomplished with the help of S. Ríos Ochoa, a bilingual and literate Maijuna individual, using a practical orthography previously established by Velie [77]. The practical orthography developed by Velie consists of 27 letters that are pronounced as if reading Spanish, with the following exceptions: in a position between two vowels, *d* is pronounced like the Spanish *r*; *ɨ* is pronounced like the Spanish *u* but without rounding or puckering the lips; and *a*, *e*, *i*, *o*, *u*, and *ɨ* are pronounced like *a*, *e*, *i*, *o*, *u*, and *ɨ* but nasalized. Also, the presence of an accent indicates an elevated tone of the voice; accents are only used when the tone is the only difference between two Maijuna words and the word’s meaning is not clarified by its context. The 27 letters that make up the Maijuna alphabet are *a*, *a*, *b*, *c*, *ch*, *d*, *e*, *e*, *g*, *h*, *i*, *i*, *j*, *m*, *n*, *ñ*, *o*, *o*, *p*, *q*, *s*, *t*, *u*, *u*, *y*, *ɨ*, and *ɨ*

^b^Transcription of Kichwa words was done by O. Coquinchi Ruiz, a bilingual and literate Kichwa individual, using an unknown orthography

^c^Low preference

^d^High preference

^e^Kichwa uses, beliefs/ceremonies, of stories

^f^Mestizo uses, beliefs/ceremonies, of stories

^g^Maijuna uses, beliefs/ceremonies, or stories

Although *A*. *seniculus* is one of the largest primates and had been eaten at some point by all of the respondents, it was not described as “tasty” by any of the interviewees, except one mestizo man. In the Maijuna culture, red howlers were infrequently eaten in the past due to food taboos or avoidance, as it was considered a sorcerer (see below). *P*. *monachus*—a medium-sized monkey—was reported as very tasty by one Maijuna interviewee. Though medium-sized and small monkey species are eaten (except for *C*. *pygmaea*), these are not frequently hunted due to their size. Many respondents stated that it is not worth using shotgun shells (due to their cost) on small primates given the limited meat obtained. Nonetheless, *L*. *nigricollis* and *A*. *vociferans* are hunted in times of game scarcity—a situation that did not occur in the area at the time of study. It is important to highlight that all of the male interviewees had hunted at some point in their lives.

All 11 primate species are occasionally raised as pets. However, respondents indicated preferences as well. *A*. *seniculus* (67%) and *C*. *albifrons* (78%) were two of the primate species with the lowest percentages of positive responses. *A*. *seniculus* are rarely kept as a pet because their loud calls disturb household members, and *C*. *albifrons* is considered mischievous and restless, making them difficult to keep as pets. *L*. *nigricollis* and *S*. *sciureus* were reported to be beneficial as pets given that they help to keep the house free of insects due to their insectivorous behavior. Among all the primate species, *L*. *lagotricha* was the most commonly reported as being kept as a pet (98%) and the only observed primate pet in the community during the study.

The tails of *P*. *monachus*, *C*. *discolor*, and *C*. *lucifer* were reported as being used as house dusters, especially those from *P*. *monachus* (48%) because of their bushy nature. Bones and teeth of *S*. *sciureus*, *C*. *discolor*, *P*. *monachus*, *C*. *albifrons*, and *A*. *seniculus* are used in handicrafts. The hyoid bone of *A*. *seniculus*, one of the most salient features of the red howler monkey, are occasionally used as a cup to drink beverages and in handicrafts. Furthermore, one male Maijuna respondent reported the use of the hyoid bone of *A*. *seniculus* to treat sore throats. According to this individual, water is poured into the hyoid bone, kept there during sunset, and ultimately consumed as a remedy. Individuals who take this remedy cannot eat red howler monkey meat anymore because they have used this primate to treat their illness. One Kichwa interviewee reported the use of the tail of *P*. *monachus* to treat digestive problems, such as diarrhea and vomiting. The tail is burned and then combined with warm water.

Among all of the uses identified, the use of most primates as sources of food and as household pets had a greater importance given a higher number of positive responses. There were no marked differences in the use of primates between Maijuna and non-Maijuna individuals, except for medicinal treatments, fur to cover drums, and the commercialization of meat (Table 4). Commercialization of primate meat (i.e., *L*. *lagotricha* and *A*. *seniculus*) was only mentioned by two interviewees, possibly indicating that primates are not frequently targeted for this activity.

### Traditional beliefs, ceremonies, and stories about primates

**Fig. 5:**
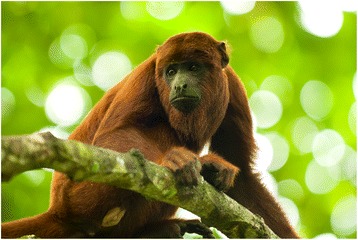
*Alouatta seniculus*, which Maijuna ancestors believed were sorcerers

Traditional beliefs regarding primates linked to dietary taboos were exclusively found among the Maijuna (Table 4). In the past, Maijuna ancestors believed that red howler monkeys (*A*. *seniculus*) were sorcerers, hence the reason why this species is sometimes called *mono brujo* in Spanish or sorcerer monkey in English (Fig. 5). According to Maijuna traditional beliefs, this monkey harmed children and adults, and its meat was avoided. Although this belief is no longer as strong as it once was, every time *A. seniculus* meat is consumed, Maijuna individuals believe that it should be done in silence as a sign of respect. In fact, if someone says: “I do not like the meat” or if it is thrown away, it is believed that a tumor may appear in the body or throat of that person, which could lead to death.

**Fig. 6:**
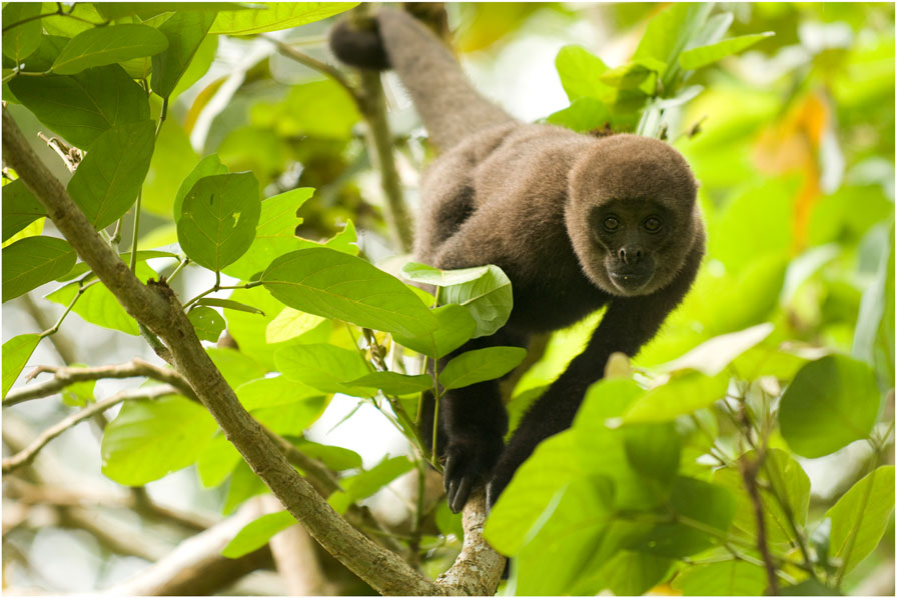
*Lagothrix lagotricha*, one of the most culturally important primates for the Maijuna

*Lagothrix lagotricha* (Fig. 6) is one of the primate species most deeply intertwined in Maijuna traditional culture. Prior to 1974, *L*. *lagotricha* was an integral part of the ceremony associated with the first yearly harvest of *Bactris gasipaes* (*pijuayo*) palm fruits. *Bactris gasipaes* is a culturally important domesticated palm, and the fruits are eaten cooked or consumed as a fermented beverage. The ceremony included the ritualistic consumption of woolly monkeys, and the meat of this monkey also served as part of a traditional courtship ritual for the Maijuna. If a woman accepted a piece of woolly monkey meat given by a Maijuna man, then she was also accepting the man, whereas if she declined the offering, it meant that she was not interested in the man and was uninterested in his proposal.

According to the Maijuna, *P*. *monachus* is considered poisonous for dogs to eat due to the “poisonous fruits” this monkey eats in the forest. Therefore, after the meat is consumed by people, any leftovers are discarded carefully. In case dogs do get poisoned, their ears are cut in order to expel the poison. The night monkey, *Aotus vociferans*, was described by some interviewees as a devil monkey that used to eat people, especially hunters in their hunting camps. This belief is related to a local name that some use for this species, which is *buri-buri*. However, most of the interviewees (58%) had never heard of the name *buri-buri*. Only 16% of interviewees agreed that *buri-buri* and the night monkey (*A*. *vociferans*) are the same animal, and 26% alleged that they are different species. Differences between the night monkey and *buri-buri* were difficult for interviewees to explain and describe due to their nocturnal behavior and the lack of visibility at night. The differences identified were mainly explained by their distinctive calls with the *buri-buri* call being described as “buri, buri, buri, buri.” The few physical differences identified between the two were (1) *buri-buri* is lighter in color than *A*. *vociferans*, (2) they have different color patterns on their foreheads, and (3) *buri-buri* is larger in size than *A*. *vociferans*. Interestingly, one Maijuna interviewee stated that *buri-buri* is in fact the Kichwa name for *A*. *vociferans*. However, this was disputed by the only Kichwa individual interviewed, who stated that the Kichwa name for *buri-buri* is in fact *makuru*. He described the *buri-buri* as a demon monkey that is smaller in size than *A*. *vociferans* with a shorter tail, lighter coloration, and yellow lines on its face.

In addition to traditional beliefs about primates, the Maijuna also have traditional stories about them. The large majority of Maijuna traditional stories associated with primates are linked to traditional cosmological beliefs as well as natural history information (e.g., physical attributes, diets, and calls of the different primate species). For the Maijuna, primates descended from humans and were created by *Maineno*, their traditional Creator, as a means to obtain fruits from trees. Recounting the transformations performed by *Maineno* helped the Maijuna to make sense of, understand, and narrate the origin of primates present in the Sucusari River basin. The creation of various monkey species and other stories are detailed in the traditional Maijuna stories presented in Appendixes 1, 2, 3, and 4. Interestingly, *C*. *pygmaea*, *A*. *vociferans*, *P*. *monachus*, and *S*. *macrocephalus* were not included in any of the Maijuna traditional stories documented as part of this study. Per the only Kichwa individual interviewed for this study, in Kichwa mythology, only *A*. *seniculus* and *C*. *discolor* have a story that details the physical attributes of both species (Appendix 5).

## Discussion

Primates are one of the most hunted taxa in Amazonia and other tropical regions around the world [47, 51, 52]. The close interaction between human and non-human primates has led to increased interest in better understanding people’s perceptions, use, and cultural beliefs of this important group of mammals. For the residents of Sucusari, how they perceive and define monkeys results in the inclusion of other arboreal mammal species beyond just primates, such as kinkajous, which is similar to ethnobiological classification systems in other parts of Amazonia [18, 21, 53, 54]. The arboreal lifestyle of these other mammal species and the fact that they can climb within and jump between trees, some of the key characteristics of Neotropical primates [55], were important factors in grouping them together with primates.

Salience of biological organisms is shaped by the degree of interactions between people and those organisms [56] and could be reflected by its cultural importance and/or its abundance in the environment. In this study, large body size, sociocultural importance, and higher abundance of certain primate species could be critical factors determining cultural salience. Salience results suggested that seven primates were particularly important to interviewees. A first group, comprised of *L*. *lagotricha*, *A*. *seniculus*, and *P*. *monachus*, are all within the top five primate species in regard to body mass in the Sucusari River basin, and they all still play an important role within Maijuna and non-Maijuna culture. A second group comprised of small-bodied primates, is made up of *S*. *sciureus*, *Callicebus* spp. and *L*. *nigricollis*. In the Sucusari River basin, *S*. *sciureus* is found in large troops (50–100; authors, personal observations) and are frequently observed along riverbanks. Considering that most of the interviewees spend large amounts of time on the river traveling and fishing, the probability of seeing troops of *S*. *sciureus* is high. A high cultural salience of *Callicebus* spp. may be linked to the fact that their calls are frequently heard in the community during the morning (authors, personal observations). And, *L*. *nigricollis* is the most abundant primate species within the river basin increasing the probability that interviewees will encounter them [36]. It is worth noting that the cultural salience of primates between Maijuna and non-Maijuna individuals in the community of Sucusari did not show marked differences among species, except for *L*. *lagotricha*, which had a salience of 0.64 for the Maijuna compared to 0.46 for non-Maijuna individuals. This result could be due to the close relationship between *L*. *lagotricha* and Maijuna cultural traditions, such as the use of *L*. *lagotricha* as an important food source and common household pet and previous use as an important ceremonial food by Maijuna ancestors.

Primates represent an important food source for Amazonian indigenous groups, and in some regions, they are considered one of the most delicious mammals [16], especially primates of the subfamily Atelinae due to their greater biomass [13, 19, 57, 58]. In the Sucusari community, primates are hunted primarily for subsistence and rarely for their commercial value (i.e., only *L*. *lagotricha* and *A*. *seniculus*). Although commercialization of primate meat does not currently represent an important source of income for the community of Sucusari, this should be closely watched given there is a market for game meat in the nearest population centers of Mazán and Iquitos.

Like many other Amazonian indigenous groups, the Maijuna used to hunt with blow guns. However, the transition to a western-style market economy allowed the Maijuna to acquire firearms, thus increasing the probability of the overexploitation of wildlife, including primates. This technology along with food preferences (i.e., particularly *L*. *lagotricha* and *P*. *monachus*) could have a negative impact on the sustainability of primate hunting in Sucusari, thus highlighting the need to determine sustainable harvest rates for these species. Large-bodied primates are more susceptible to overharvesting than other smaller primates (i.e., Callitrichid primates) due to differences in population density and lower reproductive rates [59]; therefore, they can be highly threatened by subsistence hunting if not managed effectively [14–16].

In Amazonia, primate symbolism and subsistence hunting are highly intertwined; therefore, the availability or abundance of a primate is not a direct indicator of its utilization [25]. Maijuna traditional beliefs regarding *A*. *seniculus*, considered a sorcerer monkey by Maijuna ancestors, could reflect cultural attitudes against its consumption in comparison to mestizos or Kichwa. In the past, howler monkeys created fear among Maijuna ancestors and ultimately helped to prevent overharvesting of this species. The Matsigenkas [19] and Matsés of Peru [60] as well as the Barí of Venezuela [18] claimed that howler monkeys were also avoided due to its distasteful meat and cultural taboos (i.e., possessors of spiritual hazards). Sadly, the Maijuna in the Sucusari community are currently undergoing rapid loss of their traditional beliefs [28]; thus, taboos against red howler monkey hunting are weakening and have almost disappeared (only two elder Maijuna men mentioned it during this study). Cultural taboos associated with red howler monkeys are an important tool to counteract the effects of subsistence hunting, which could lead to unsustainable harvest yields on howler populations. Cultural beliefs associated with *A*. *vociferans* and *buri-buri* (the demon monkey) remain unclear, creating uncertainty around the origin of this belief. Two main possibilities exist: (1) *buri-buri* is a non-Maijuna belief that has been learned and incorporated only by some individuals in the community or (2) it is a Maijuna belief that is being lost due to the rapid expansion of westernized culture.

The capture of primate infants as pets is commonly reported in the Amazon as an offshoot of hunting [24, 57]. It is done through seeking female primates with their infants and retrieving them after the mother is shot and falls to the ground. Selective hunting of females may represent a challenge for the sustainability of primate populations and subsistence hunting in the community of Sucusari, leading to skewed sex ratios in primate populations, which is exacerbated by their low reproductive rates [61]. Ateline primates are of particular concern given that they are considered preferred primate prey as well as being reported as favorite pets among several Amazonian indigenous communities [19, 25, 58]. Pet keeping also holds a particular significance for indigenous groups [20]. For the Guajá, keeping primates as pets plays a key role in defining the socialization of children, such as developing infant care for girls and hunting skills for boys [20]. During this study, woolly monkeys were the only primates being kept as pets by one Maijuna woman in the village, who was not able to give birth. The two woolly monkeys were treated like family members. She gave them a traditional alcoholic beverage brewed from cassava, treated them with medicines if they had malaria or other illnesses, and gave them human names. It is worth noting that although only two woolly monkeys were observed as pets during this study, a variety of other primate species have been observed as pets over the years in the Maijuna communities (authors, personal observations).

The wide variety of uses of primates, especially for food, folk medicine, and pet keeping, among Amazonian communities have serious implications for human health [62], which are rarely discussed or taken into account while studying human and non-human primate interactions. Zoonotic diseases such as malaria, dengue, leishmaniasis, leptospirosis, etc. are some of the most common in the Neotropics [63] and are all found in the Sucusari community (authors, personal observations). The consumption of primates for food or medicinal purposes aids in the spread of pathogenic organisms to humans. Thus, special attention should be given while assessing primate meat consumption, pet keeping, or traditional medicines, due to the fact that these species could be potential reservoirs for pathogen transmission, particularly with the increased interest in potential zoonotic malaria. Recently, a study conducted in southern Peru identified two primate hosts (*Saguinus imperator* and *Saguinus fuscicollis*) for *Plasmodium brasiliensis* and *Plasmodium malariae*, the latter being the one that infects humans [64]. Furthermore, this research suggested and provided evidence that Callitrichids might act as reservoirs for human zoonotic malaria. In the community of Sucusari, efforts to test non-human primates that are in contact with humans for the presence of the protozoan parasite that causes malaria are needed to assess the possibility of zoonoses.

For indigenous people, traditional stories symbolize unity and the creation of shared bonds [65]. Most of these stories document the creation of their lands, disentangle natural events, and/or provide the ethical and moral foundations on which indigenous cultures are built [66, 67]. The traditional stories about primates of the Maijuna and other Amazonian indigenous groups [18, 19] explain their origin through the transformation of human beings into monkeys by their creators. These stories highlight the significance of natural resources for the survival of living beings, reinforcing the connection indigenous people have with nature [68, 69]. The Maijuna monkey creation story is rooted in Maijuna cosmological beliefs and symbolizes the relationship between humans and nonhuman primates, including detailed natural history information.

For the Maijuna, an increase in contact with outsiders and missionaries, a western education system that does not value traditional knowledge, and the entry of mestizos into their communities and their integration into the market economy has led to a disconnect with their cultural practices, causing a rapid loss of their traditional knowledge [28, 29], commonly recognized as acculturation [70]. Unfortunately, in the community of Sucusari, acculturation is manifested in younger generations, where cultural knowledge—especially the Maijuna language and traditional beliefs (e.g., cultural taboos associated with red howler monkeys), ceremonies (e.g., the first yearly harvest of *Bactris gasipaes* fruits), and stories (e.g., the Maijuna monkey creation story)—is being lost at a rapid rate [28].

The importance of the role of indigenous people in conservation is increasing [71–74]. This is especially true in the case of the Maijuna given the recent creation of the immense and heavily forested MKRCA. In the community of Sucusari, despite the effects of acculturation on the traditional lives of the Maijuna, community members still remain deeply connected to their ancestral lands and rely heavily on natural resources for both subsistence and income generation. The results of this study highlight that primates are regularly hunted and culturally significant in a wide variety of ways to the community. Additionally, some species hold special cultural value for the Maijuna. All of this information helps to shed light on the ethnoprimatology of the Maijuna and can help to focus conservation efforts on primate species of particularly high sociocultural importance as well as ecological value, such as *L*. *lagotricha*. Ultimately, this has implications for working to sustainably manage hunting within the Sucusari river basin and, thus, on the viability of primate populations.

## Conclusion

Understanding the sociocultural importance and use of primates by indigenous and local communities should be an essential component of any primatological study and should not be overlooked. This research highlights that primates are regularly hunted and culturally significant in a wide variety of ways to the community of Sucusari. It has shed light on culturally important primate species, particularly for the Maijuna, and how the rapid loss of traditional knowledge, beliefs, and practices may have a negative impact on primate populations and Maijuna cultural identity. Given that biologists frequently ignore the existence of traditional knowledge and beliefs within primatological studies, understanding their significance is critical to identify cultural factors that influence hunter behavior and choice and the concomitant effects on primate populations. Information on what primate species are most frequently hunted and how they are used, what percentage of game meat consists of primates, the evaluation and monitoring of primate hunting levels (including harvest rates), and whether there are zoonotic infections from primates in the community should be a priority to ensure the long-term conservation of primates as well as the protection of human welfare.

Although the Maijuna are interested in the implementation of sustainable management practices, their success will depend on local circumstances and buy-in from the entire community, including non-Maijuna individuals. The community of Sucusari represents the current situation in many Amazonian indigenous communities, where a mix of different ethnic groups inhabits the same land. Therefore, the understanding of sociocultural differences in regards to resource use among different ethnic groups living in a single area is critical for effective community engagement and community-based primate conservation programs. In the context of the recently created MKRCA, the actions of both Maijuna and non-Maijuna individuals in the Sucusari community can have profound consequences on the primate populations of the area. Therefore, the effective conservation of the MKRCA and its primates will depend on the capacity of individuals from different backgrounds and cultural traditions to successfully work together. More specifically, the success of primate conservation will hinge on their willingness to make decisions that have positive, beneficial, and lasting impacts on both forest protection and livelihoods.

Managing hunting in the MKRCA should be community-based and community-driven. Thus, ensuring full participation and involvement of all community members is absolutely critical. Otherwise, any restrictions on the hunting of primates may not be welcome nor completely adhered to. In short, given the biological and cultural value of the MKRCA, the main role of outside stakeholders (e.g., scientists, conservation organizations, and government agencies) should be to help empower the Maijuna and other residents of their communities, through scientific knowledge and monitoring techniques (e.g., hunting registers, biological surveys, etc.), to implement community-based management plans that not only support the conservation goals of the protected area but also their cultural and economic needs. In conclusion, this study stresses the need of integrating an ethnoprimatological approach in primate conservation; ultimately integrating the goals of biodiversity conservation with the cultural and economic needs of indigenous and local communities.

## Appendix 1

English translation of the traditional Maijuna monkey creation story. Story told by Samuel Ríos Flores, a master Maijuna storyteller. The Maijuna version of this story is presented in Appendix 2. The numbered sentences in the English version of this story correspond exactly to the Maijuna version.

^1^“We want to eat fruits,” [said the group of people]. ^2^“You want to eat fruits?” [asked the Creator]. ^3^“Yes, we wish we could go up and eat them,” [replied the group of people]. ^4^“If you want fruits then untie the rope from your hammock and place it down toward your butt (to make a tail),” [said the Creator]. ^5^[In the meantime] the Creator was grating *Genipa americana* fruits. ^6^After grating the fruit he rubbed it on their faces and mouths (*Genipa americana* fruits are used to make a black dye and, according to Maijuna traditions, this is why *Lagothrix lagotricha* monkeys have black faces.) ^7^“Who wants to be a *naso* (*L. lagotricha*)?” [he asked]. ^8^“I do,” [a woman replied]. ^9^“What does a *naso* (*L. lagotricha*) sound like while eating fruits?” [asked the Creator]. ^10^She listened to him, climbed a tree, and said: “chichi, chichi, chichi.” ^11^“You are a *chichi* (*L. nigricollis*). ^12^You are not a real *naso* (*L. lagotricha*),” he said. ^13^“This is what I will be then,” the women said. (At this moment she became a *chichi* monkey.) ^14^“Who is going to be a *naso* (*L. lagotricha*)?” [the Creator asked again]. ^15^“Me,” [replied a man]. ^16^“What does a real *naso* (*L. lagotricha*) sound like?” [asked the Creator]. ^17^The man climbed a tree and happily gathered fruits. ^18^[He then called out], “Choyoro, choyoro, choyoro.” ^19^“Yes, you are a *naso* (*L. lagotricha*),” [said the Creator]. ^20^“Now, who will be a *jaiqu*ɨ (*Alouatta seniculus*)?” [asked the Creator]. ^21^“Me. ^22^I want to be a *jaiqu*ɨ (*A. seniculus*),” [a women replied]. ^23^“Let me hear you. ^24^Sing so I can hear how a *jaiqu*ɨ (*A. seniculus*) sings,” [requested the Creator]. ^25^“Oju, oju, oju, oju,” [she sang]. ^26^“You are a *bao* (*Callicebus lucifer*)”, [said the Creator]. ^27^“This is what I will be then,” [replied the woman]. ^28^“Now, who will be a *jaiqu*ɨ (*A. seniculus*)?” [asked the Creator]. ^29^“Me,” [a man replied]. ^30^The creator then placed a small *bichib*ɨ (gourd) into the man’s throat. ^31^Then, the creator rubbed annatto over his entire body (to paint it red). ^32^“Sing so I can hear if you are really a *jaiqu*ɨ (*A. seniculus*). ^33^How will you sing when it rains?” [asked the Creator]. ^34^“Ogu, ogu, ogu, oguuu,” [he howled]. ^35^“Yes, you are a *jaiqu*ɨ (*A. seniculus*),” [said the Creator]. ^36^The end.

## Appendix 2

Maijuna version of the traditional Maijuna monkey creation story. Story told by Samuel Ríos Flores, a master Maijuna storyteller.

^1^*Acue acueyo oiyi yiqu*ɨ*a*. ^2^*M*ɨ*sa acue oiye*. ^3^*Quima m*ɨ*ni acueyo oiyi*. ^4^*Acue oij*ɨ *ani m*ɨ*sa* *j**a*ɨ*oma josema m*ɨ*sajuna bari oje t**a**tecachi*. ^5^*Be* ɨ*r*ɨ*gu*ɨ. ^6^*Be* ɨ*r*ɨ*re yia qu*ɨ*r*ɨ*gu*ɨ *yob**ɨ* *tea*. ^7^*Neb*ɨ *naso bayo* *i**j*ɨ. ^8^*Yi bachi* *i**co*. ^9^*Quima j**ɨ**caqu*ɨ *naso acue acuequ*ɨ *ani*. ^10^*Asare m*ɨ*ico chi*, *chi*, *chi*, *chi* *i**co*. ^11^*Chichina j**a*. ^12^*A**je nasona j**a* *i**j*ɨ. ^13^*C**ao* *ñi bachi* *i**co*. ^14^*Neb*ɨ *bayo*. ^15^*Yia* *i**j*ɨ. ^16^*Quima j*ɨ*caqu*ɨ *naso deb*ɨ *ani*. ^17^*M*ɨ*ni acue t*ɨ*tequ*ɨ *chibaj*ɨ. ^18^*Choyoro*, *choyoro*, *choyoro*. ^19^*Ase c**aɨ**ta nasona chibaj*ɨ. ^20^*Jana* *i**gueca ne bayo jaiqu*ɨ. ^21^*Yia* *i**co*. ^22^*Yi jaiqu*ɨ *bachi*. ^23^*J**a* *j*ɨ*cama asayi*. ^24^*J**a* *yima asayi jaiqu*ɨ *quima yiqu*ɨ. ^25^*Oju*, *oju*, *oju*, *oju*. ^26^*Baonata*. ^27^*C**ao**ñi bachi* *i**co*. ^*28*^*Jana* *i**gueca ne bayo jaiqu*ɨ. ^29^*Yia* *i**j*ɨ. ^30^*Bichib*ɨ *t**a**tecaqu*ɨ. ^31^*Bosa s**o**caqu*ɨ. ^32^*J**a* *yima asayi jaiqu*ɨ *ani*. ^33^*Ocotu quima yiqu*ɨ *bachi*. ^34^*Ogu*, *ogu*, *ogu*, *oguuu*. ^35^*Ase c**aɨ**ta* *jaiqu*ɨ*na j**a* *chibaj*ɨ. ^36^*C**a**s**oa* *j**a*.

## Appendix 3

English translation of the traditional Maijuna story of the red titi monkey (*Callicebus discolor*). Story told by Romero Ríos Ochoa.

There was a lazy man, who never wanted to work or walk. For him it was a problem and he didn’t know what was a matter. A wise person came and told him, “You have some animals in your body.” The man asked, “How can we kill these animals?”

“We are going to offer them fruits (*Annona spp*.) so that they come down to eat. We will kill them when they come down to eat and you will become a hard-working person,” answered the wise man. When the fruits were set down, monkeys descended from the man’s shoulders and started to eat the fruits. However, one monkey didn’t go down, it stayed inside the man’s body. The rest of the monkeys were killed by the man.

With fewer burdens on his body, the man began to work and he was no longer lazy. All of these animals were *ñame bao* (*Callicebus discolor*), for this reason they are called the “lazy monkeys.” The end.

## Appendix 4

English translation of the traditional Maijuna story of the common squirrel monkey (*Saimiri sciureus*). Story told by Felipe Navarro Rojas.

The people were looking at the trees (*Inga* spp.). They really wanted to eat the fruits. They asked themselves, “How can we reach the fruits?” At this moment, *Maineno* appeared. “What are you talking about?” asked *Maineno*. “We want to eat the fruits but we are not able to”, replied the group of people. *Maineno* then let them climb the trees and said, “Now you will all be *bo chichi* (*Saimiri sciureus*).” That is how *bo chichi* were created.

## Appendix 5

English translation of the traditional Kichwa story of the red titi monkey (*Callicebus discolor*) and red howler monkey (*Alouatta seniculus*). Story told by Orlando Coquinche Ruiz.

The red titi monkey (*Callicebus discolor*) had its little drum (referring to the modified hyoid bone used for vocalization) similar to the red howler monkey (*Alouatta seniculus*). The drum of the red titi monkey was louder [than that of the red howler monkey]. One day, the red titi monkey lent his drum to the red howler monkey because the howler’s was not producing a strong sound. After lending his drum to the red howler monkey, the red howler monkey never gave it back. For this reason, the red titi monkey no longer sings loud and now the red howler monkey beats it at singing and sings much louder.

## Abbreviation

MKRCA: Maijuna-Kichwa Regional Conservation Area

## Footnotes

1 *Mestizos* are individuals of mixed Amerindian and Iberian descent who live throughout the Peruvian Amazon region and practice a mixture of traditional agriculture, hunting, fishing, and forest product extraction for their livelihoods [75].
